# Identification of the clinically most relevant postoperative complications after gastrectomy: a population-based cohort study

**DOI:** 10.1007/s10120-019-00997-x

**Published:** 2019-09-03

**Authors:** Emma C. Gertsen, Lucas Goense, Hylke J. F. Brenkman, Richard van Hillegersberg, Jelle P. Ruurda, the Dutch Upper Gastrointestinal Cancer Audit (DUCA) group

**Affiliations:** 1 Division Cancer Center, Department of Surgery, University Medical Center Utrecht, Utrecht University, Heidelberglaan 100, 3508 GA Utrecht, The Netherlands; 2 Department of Radiation Oncology, University Medical Center Utrecht, Utrecht University, Utrecht, The Netherlands

**Keywords:** Gastric cancer, Gastrectomy, Morbidity, Population-based

## Abstract

**Background:**

Postoperative complications frequently occur after gastrectomy for gastric cancer and are associated with poor clinical outcomes, such as mortality and reoperations. The aim of study was to identify the clinically most relevant complications after gastrectomy, using the population-attributable fraction (PAF).

**Methods:**

Between 2011 and 2017, all patients who underwent potentially curative gastrectomy for gastric adenocarcinoma were included from the Dutch Upper GI Cancer Audit. Postoperative outcomes (morbidity, mortality, recovery and hospitalization) were evaluated. The prevalence of postoperative complications (e.g., anastomotic leakage and pneumonia) and of the study outcomes were calculated. The adjusted relative risk and Confidence Interval (CI) for each complication-outcome pair were calculated. Subsequently, the PAF was calculated, which represents the percentage of a given outcome occurring in the population, caused by individual complications, taking both the relative risk and the frequency in which a complication occurs into account.

**Results:**

In total, 2176 patients were analyzed. Anastomotic leakage and pulmonary complications had the greatest overall impact on postoperative mortality (PAF 29.2% [95% CI 19.3–39.1] and 21.6% [95% CI 10.5–32.7], respectively) and prolonged hospitalization (PAF 12.9% [95% CI 9.7–16.0] and 14.7% [95% CI 11.0–18.8], respectively). Anastomotic leakage had the greatest overall impact on re-interventions (PAF 25.1% [95% CI 20.5–29.7]) and reoperations (PAF 30.3% [95% CI 24.3–36.3]). Intra-abdominal abscesses had the largest impact on readmissions (PAF 7.0% [95% CI 3.2–10.9]). Other complications only had a small effect on these outcomes.

**Conclusion:**

Surgical improvement programs should focus on preventing or managing anastomotic leakage and pulmonary complications, since these complications have the greatest overall impact on clinical outcomes after gastrectomy.

**Electronic supplementary material:**

The online version of this article (10.1007/s10120-019-00997-x) contains supplementary material, which is available to authorized users.

## Introduction

Gastrectomy with lymphadenectomy is the cornerstone of curative treatment for patients diagnosed with adenocarcinoma of the stomach [1] and, if deemed fit enough, patients will also receive perioperative chemotherapy [2]. Gastrectomy is associated with a high risk of postoperative complications (up to 40%) [3], of which pulmonary complications, anastomotic leakage, and wound complications occur most frequently [4–9]. These complications have a negative influence on postoperative outcomes, such as mortality, length of hospital stay, number of reoperations, and readmissions [10, 11]. The effects of various complications on deteriorated postoperative outcomes also result in a significant increase in healthcare costs [12, 13].

Identification of complications that have the most effect on outcomes after gastrectomy is important for efficient allocation of healthcare resources. Yet, few studies have explicitly measured the population burden of complications on outcomes after gastrectomy. In this context, the population-attributable fraction (PAF) is a useful measure, because it represents the anticipated percentage reduction of a given outcome in case a certain complication would be completely prevented [14]. The strength of the association of a complication with an outcome is represented by the relative risk (RR). However, the RR does not reckon with the frequency in which a complication occurs, whereas the PAF takes both frequency and the relative risk into account [15, 16]. The introduction of centralization of specialized care across several countries in Europe has led to an overall reduction in the number of complications [17–21]. Using the PAF to describe the impact of postoperative complications will be of direct relevance for prioritizing research agendas and acquirement of appropriate funding, primary prevention efforts, and resource allocation to enhance reduction of postoperative complications after gastrectomy. Consequently, the aim of the current study was to assess the impact of relevant postoperative complications on predefined outcomes and to subsequently identify the clinically most relevant postoperative complications after gastrectomy for gastric cancer as measured by the PAF.

## Methods

### Study design

This population-based cohort study included data from a prospective nationwide registration of all patients that underwent a surgical resection for gastroesophageal cancer, the Dutch Upper GI Cancer Audit (DUCA). All hospitals that perform gastric surgery in The Netherlands are obliged to annually provide data on patient and tumor characteristics, items regarding processes of care, and clinical and pathological outcomes of surgery. Being part of the Dutch Institute for Clinical Auditing (DICA) that organizes national audits in a uniform format, DUCA provides complete and reliable registered data, as was reported by an independent team of data managers that performed an in-depth quality investigation [3]. The scientific committee of the DUCA approved the current study, and no ethical approval or informed consent was required under Dutch law.

### Patient population and treatment

Between 2011 and 2017 all patients with gastric adenocarcinoma (cT1-4a-x, N0-3-x, M0-x) who underwent elective (sub)total gastrectomy with curative intent were selected from the DUCA. Curative treatment consisted of a (sub)total gastrectomy with, in case of an advanced disease stage, a modified D2 (D1+) lymphadenectomy (without pancreaticosplenectomy and corresponding lymph node station 10), according to the Japanese Gastric Cancer Treatment Guidelines [22]. According to these guidelines, lymph node stations 1, 3, 4d, 4sb, 5-9, 11p, and 12a are dissected in case of distal gastrectomy and lymph node stations 1–3, 4d, 4sa, 4sb, 5–9, 11p, 11d, and 12a are dissected during total gastrectomy. Furthermore, if patients with an advanced tumor (cT2+ or cN+) were considered fit enough, perioperative chemotherapy according or comparable to the MAGIC trial regimen was offered [2, 23]. If during surgery, no Roux-en-Y or Billroth reconstruction was created, or no lymphadenectomy was performed, patients were excluded. According to the Dutch national guidelines, patients were staged using gastroscopy and computed tomography (CT) of the thorax and abdomen [24] and since their recent implementation in July 2016, with ^18^F fluorodeoxyglucose (FDG) positron emission tomography (PET)/CT and staging laparoscopy in case of an advanced tumor (cT3-4, cN+) [1]. Tumors were classified according to the 7th edition of the American Joint Committee on Cancer TNM staging system [25].

### Predictors and study outcomes

Postoperative morbidity was appraised and divided into four groups: intra-abdominal complications (including anastomotic leakage, abscesses, and bleeding), wound complications, non-surgical complications (including pulmonary, cardiac, thromboembolic, neurological, and urological complications), and other complications. Definitions of the abovementioned complications are given in Table 2. The study outcomes included mortality (defined as death during the initial hospital stay or within 30 days after surgery), prolonged hospitalization (defined as hospital stay that exceeds the 75th percentile value), re-interventions (consisting of radiological/endoscopic/surgical interventions), reoperation (defined as a postoperative surgical procedure under general anesthesia), and readmission (within 30 days after initial discharge). All complications were scored according to the standards of the DUCA, provided via online information [26].

### Statistical analysis

Patient and treatment-related characteristics are described as mean ± standard deviations (SD) and categorical data are presented as frequencies (percentages). Missing information of patients for one or more variables was imputed with the iterative Markov chain Monte Carlo method (5 iterations) [27]. The frequency of initial missing’s per variable is presented in Table 1. All statistical analyses were performed using SPSS version 24.0 (IBM Corp., Armonk, NY) and R language environment (version 3.3.1, http://www.R-project.org, ‘geeglm’, ‘sandwich’, ‘mice’, and ‘AF’ packages). Significance was set at *p < *0.05.

**Table 1:** Baseline characteristics of 2176 patients who underwent elective, intentionally curative gastrectomy for gastric cancer

	No. (%)	Initial missing values (%)
Patient characteristics		
Age, years (mean ± SD)	68.6 ± 11.7	1 (< 1%)
BMI, kg/m^2^ (mean ± SD)	25.2 ± 4.5	44 (2%)
Gender (% male)	1343 (62)	0 (0%)
ASA-classification		10 (1%)
I	303 (14)	
II	1225 (56)	
III	627 (29)	
IV	21 (1)	
Comorbidities		0 (0%)
Asthma/COPD	270 (12)	
Coronary artery disease^a^	253 (12)	
History of myocardial infarction	175 (8)	
History of arrhythmia	302 (14)	
Hypertension	763 (35)	
Peripheral vascular disease	101 (5)	
Diabetes mellitus	368 (17)	
History of CVA	103 (5)	
History of thromboembolic events	157 (7)	
Endocrine disorder	131 (6)	
Previous abdominal or thoracic surgery	868 (40)	0 (0%)
Tumor characteristics		
cT-stage		515 (23%)
T1	202 (9)	
T2	675 (31)	
T3	1169 (54)	
T4	130 (6)	
cN-stage		235 (11%)
N0	1287 (59)	
N1	583 (27)	
N2	192 (9)	
N3	44 (2)	
Nx	70 (3)	
Treatment characteristics		
Neoadjuvant treatment	1339 (62)	3 (< 1%)
Resection type		0 (0%)
Total gastrectomy	905 (42)	
Subtotal gastrectomy	1271 (58)	
Surgical approach (% open procedure)	1352 (62)	0 (0%)
Conversion	87 (4)	0 (0%)
Tumor location		53 (2%)
Fundus	165 (8)	
Corpus	691 (32)	
Antrum	959 (44)	
Pylorus	189 (9)	
Whole stomach	98 (5)	
Other	74 (3)	

Percentages may not add up to 100% due to rounding

*ASA* American Society of Anesthesiologists, *BMI* body mass index, *COPD* chronic obstructive pulmonary disease, *CVA* cerebrovascular accident

^a^Patients with a history of angina pectoris, percutaneous transluminal coronary angioplasty (PTCA) and/or coronary artery bypass graft (CABG)

### Population-attributable fraction (PAF)

As stated in the introduction, the PAF is a useful measure to present the impact of a complication, as it takes both frequency and the relative risk of a certain outcome into account [15, 16]. To determine the PAF, first, the prevalence of complications and study outcomes was calculated. The adjusted relative risk (aRR) and Confidence Interval (CI) for each complication-outcome pair were calculated using multivariable Poisson regression models with log link and robust error variance. The PAF was calculated with the AF package in R software which allows for confounder-adjusted estimation of PAFs for cohort studies [28]. The models were adjusted for patient and treatment-related characteristics (i.e., age, gender, Body Mass Index, American Society of Anesthesiologists (ASA) score, comorbidities, previous abdominal or thoracic surgery, use of immunosuppressant drugs, open or minimally invasive surgery, total or partial gastrectomy, conversions, cTN stage, and neoadjuvant treatment) and for all complications with a significant association with the study outcome (to adjust for simultaneous occurrence of coexisting complications and thereby prevent overestimation of the individual contribution of a specific complication). The severity of a complication (as assessed by the Clavien–Dindo classification) was not integrated in the analysis, since this would have incorporated the outcomes of our study (which indirectly define the severity of the complication, e.g., re-intervention, mortality) into our determinants (i.e., complications).

In this study, the risk-adjusted PAF represents the anticipated percentage reduction of a given outcome (i.e., mortality, prolonged hospital stay, reoperation, and readmission) in case a certain complication would be completely prevented in our study population.

## Results

### Study population

In The Netherlands, 2304 patients underwent an elective (sub)total gastrectomy with curative intent for primary gastric adenocarcinoma during the study period. Some 128 of these patients were excluded, as no lymphadenectomy was performed or no Roux-Y or Billroth reconstruction was made. Of the remaining 2176 patients, 1343 (62%) were male and the mean age was 68.6 (± 11.7) years. The majority of patients had an ASA score of 2 (56%), and hypertension (35%) and diabetes mellitus (17%) were the most common comorbidities. Most patients had a cT3 tumor (54%), cN0-stage (59%), and were treated with neoadjuvant therapy (62%). Patient and treatment-related characteristics are presented in Table 1.

### Postoperative outcomes

All postoperative complications and clinical outcomes are shown in Table 2. Pulmonary complications (15%), anastomotic leakage (7%), and cardiac complications (6%) were the most common complications. Postoperative mortality occurred in 118 patients (5%), prolonged hospitalization in 484 patients (22%), reoperations in 247 patients (11%), and 259 patients (12%) was readmitted. Ileus occurred in 4% of patients, but was only scored in DUCA since 2016 and, therefore, not shown in Table 2.

**Table 2:** Postoperative complications and clinical outcomes after elective gastrectomy of 2176 patients

	No. (%)	Initial missing values^l^
Postoperative complications		
Pulmonary complication^a^	321 (15)	0
Anastomotic leakage^b^	154 (7)	0
Cardiac complication^c^	122 (6)	0
Acute delirium	103 (5)	0
Abscess	85 (4)	0
Wound infection	93 (4)	0
Urological complication^d^	80 (4)	0
Thromboembolic complication^e^	32 (2)	0
Chyle leakage	38 (2)	0
Postoperative bleeding	35 (2)	0
Bowel injury	26 (1)	0
Pancreatitis	10 (< 1)	0
Clinical outcomes		
Postoperative mortality^f^	118 (5)	0
Duration of hospital stay (days)^g^	9 (7–13)	17
Prolonged hospitalization^h^	484 (22)	17
Re-intervention^i^	344 (16)	0
Reoperation^j^	247 (11)	0
Hospital readmission^k^	259 (12)	0

Values in parentheses are percentages unless indicated otherwise. Data shown in Table represent the dataset after imputation

^a^Pneumonia, pleural effusion, respiratory failure, pneumothorax and/or acute respiratory distress syndrome (ARDS)

^b^Any clinically or radiologically proven anastomotic leakage

^c^Supra- and ventricular arrhythmia, myocardial infarction and/or heart failure

^d^Acute renal insufficiency, acute kidney failure requiring dialysis, urine tract infection and/or urine retention

^e^Pulmonary embolism, deep venous thrombosis and/or cerebro vascular accident

^f^Death during initial hospital admission or within 30 days after surgery

^g^Data are depicted as median (IQR)

^h^Length of hospital stay ≥75th percentile (for each surgical approach)

^i^Re-intervention (radiological/endoscopic/surgical)

^j^Postoperative surgical procedure under general anesthesia

^k^Readmission to hospital within 30 days after initial discharge

^l^Number of missing values for each variable before imputation

The risk-adjusted associations between the postoperative complications and subsequent clinical outcomes are described in Tables 3, 4, 5, 6, and 7. Anastomotic leakage and bowel injury were associated with the greatest relative risk of both postoperative mortality and reoperations (aRR 6.32 [95% CI 4.18–9.49] and aRR 8.56 [95% CI 6.46–11.3] for anastomotic leakage, respectively, and aRR 5.27 [95% CI 2.27–10.67] and aRR 8.03 [95% CI 5.03–12.30] for bowel injury, respectively). Anastomotic leakage was associated with the greatest relative risk for prolonged hospitalization (aRR 3.94 [95% CI 3.12–4.93]), followed by intra-abdominal abscess (aRR 3.57 [95% CI 2.67–4.69]). In fact, all postoperative complications were significantly associated with prolonged hospitalization. All postoperative complications, except urological complications, were associated with re-interventions, with anastomotic leakage (aRR 7.25 [95% CI 5.70–9.21]) and intra-abdominal abscesses (aRR 6.22 [95% CI 4.73–8.12]) having the highest association. Intra-abdominal abscesses and bowel injury were associated with the greatest risk of hospital readmission (aRR 3.24 [95% CI 2.16–4.70] and 2.93 [95% CI 1.38–5.45], respectively).

**Table 3:** Risk-adjusted associations and population-attributable fraction between postoperative mortality and complications after elective resection for gastric cancer

Postoperative complication	No. died/survived (% died)	Risk-adjusted association^a^		Risk-adjusted PAF^b^	
		Adjusted relative risk (95%CI)	*p* value	PAF% (95% CI)	*p* value
Pulmonary complication	49/272 (15)	3.02 (2.06–4.41)	< 0.001	21.6 (10.5–32.7)	< 0.001
Anastomotic leakage	45/109 (29)	6.32 (4.18–9.49)	< 0.001	29.2 (19.3–39.1)	< 0.001
Cardiac complication	25/97 (21)	3.10 (1.91–4.87)	< 0.001	8.9 (1.1–1.7)	0.025
Acute delirium	14/89 (14)	1.71 (0.92–2.95)	0.054	–	–
Abscess	11/74 (13)	2.30 (1.15–4.16)	0.003	0.3 (− 5.2–5.9)	0.902
Wound infection	10/83 (11)	1.64 (0.79–3.02)	0.119	–	–
Urological complication	8/72 (10)	1.41 (0.62–2.76)	0.362	–	–
Thromboembolic complication	5/27 (16)	2.82 (0.97–6.49)	0.034	0.2 (− 3.7–4.1)	0.921
Chyle leakage	2/36 (5)	0.93 (0.15–2.97)	0.911	–	–
Post–operative bleeding	7/28 (20)	3.53 (1.47–7.20)	< 0.001	2.7 (− 1.1–6.6)	0.162
Bowel injury	8/18 (31)	5.27 (2.27–10.67)	< 0.001	4.2 (0.2–8.2)	0.036

Death during initial hospital admission or within 30 days after surgery

*PAF* population-attributable fraction

^a^Multivariable Poisson regression

^b^Logistic regression-based estimates of confounder-adjusted attributable fractions

**Table 4:** Risk-adjusted associations and population-attributable fraction between prolonged hospitalization and complications after elective resection for gastric cancer

Postoperative complication	No. with/without prolonged stay (% prolonged)	Risk-adjusted association^a^		Risk-adjusted PAF^b^	
		Adjusted relative risk (95% CI)	*p* value	PAF% (95% CI)	*p* value
Pulmonary complication	162/159 (50)	2.78 (2.29–3.38)	< 0.001	14.7 (11.0–18.8)	< 0.001
Anastomotic leakage	112/42 (73)	3.94 (3.12–4.93)	< 0.001	12.9 (9.7–16.0)	< 0.001
Cardiac complication	62/60 (51)	2.22 (1.67–2.91)	< 0.001	2.8 (0.4–5.2)	0.021
Acute delirium	52/51 (51)	2.26 (1.66–3.01)	< 0.001	2.6 (0.6–4.4)	0.008
Abscess	59/26 (69)	3.57 (2.67–4.69)	< 0.001	5.3 (3.2–7.5)	< 0.001
Wound infection	58/35 (62)	2.96 (2.20–3.89)	< 0.001	4.6 (2.8–6.9)	< 0.001
Urological complication	31/49 (39)	1.59 (1.08–2.26)	0.003	1.3 (− 0.3–2.9)	0.121
Thromboembolic complication	17/15 (53)	2.63 (1.54–4.17)	< 0.001	0.8 (− 0.2–1.8)	0.145
Chyle leakage	17/21 (45)	2.16 (1.27–3.42)	< 0.001	1.6 (0.4–2.8)	0.006
Postoperative bleeding	19/16 (54)	2.29 (1.39–3.53)	< 0.001	1.4 (0.2–2.6)	0.015
Bowel injury	18/8 (69)	2.98 (1.78–4.68)	< 0.001	1.2 (0.0–2.3)	0.052

Length of hospital stay ≥75th percentile (for each surgical approach)

*PAF* population-attributable fraction

^a^Multivariable poisson regression

^b^Logistic regression-based estimates of confounder-adjusted attributable fractions

**Table 5:** Risk-adjusted associations and population-attributable fraction between re-interventions and complications after elective resection for gastric cancer

Postoperative complication	No. with/without re-intervention (% re-intervention)	Risk-adjusted association^a^		Risk-adjusted PAF^b^	
		Adjusted relative risk (95% CI)	*p* value	PAF% (95% CI)	*p* value
Pulmonary complication	119/202 (37)	2.72 (2.15–3.42)	< 0.001	11.3 (7.2–15.5)	< 0.001
Anastomotic leakage	129/25 (84)	7.25 (5.70–9.21)	< 0.001	25.1 (20.5–29.7)	< 0.001
Cardiac complication	46/76 (38)	2.36 (1.69–3.23)	< 0.001	1.6 (– 0.8–4.0)	0.206
Acute delirium	37/66 (36)	2.29 (1.58–3.21)	< 0.001	1.7 (– 0.6–3.9)	0.153
Abscess	72/13 (85)	6.22 (4.73–8.12)	< 0.001	11.5 (8.2–14.8)	< 0.001
Wound infection	57/36 (61)	4.28 (3.15–5.73)	< 0.001	6.9 (4.4–9.9)	< 0.001
Urological complication	20/60 (25)	1.50 (0.92–2.32)	0.050	–	–
Thromboembolic complication	13/19 (41)	2.61 (1.41–4.40)	< 0.001	0.2 (– 0.6–0.9)	0.674
Chyle leakage	13/25 (34)	2.28 (1.23–3.83)	< 0.001	1.5 (0.2–2.9)	0.029
Post–operative bleeding	26/9 (74)	4.60 (2.99–6.79)	< 0.001	4.1 (2.0–6.3)	< 0.001
Bowel injury	25/1 (96)	6.02 (3.85–9.01)	< 0.001	3.6 (1.8–5.5)	< 0.001

Re-intervention (radiological/endoscopic/surgical)

*PAF* population-attributable fraction

^a^Multivariable poisson regression

^b^Logistic regression-based estimates of confounder-adjusted attributable fractions

**Table 6:** Risk-adjusted associations and population-attributable fraction between reoperation and complications after elective resection for gastric cancer

Postoperative complication	No. with/without reoperation (% reoperation)	Risk-adjusted association^a^		Risk-adjusted PAF^b^	
		Adjusted relative risk (95% CI)	*p* value	PAF% (95% CI)	*p* value
Pulmonary complication	85/236 (27)	2.62 (1.98–3.43)	< 0.001	10.3 (4.8–15.9)	< 0.001
Anastomotic leakage	102/52 (66)	8.56 (6.46–11.3)	< 0.001	30.3 (24.3–36.3)	< 0.001
Cardiac complication	33/89 (27)	2.37 (1.59–3.42)	< 0.001	2.2 (– 0.1–5.7)	0.224
Acute delirium	24/79 (23)	2.04 (1.29–3.08)	< 0.001	0.4 (– 2.6–3.5)	0.787
Abscess	41/44 (48)	4.75 (3.32–6.66)	0.003	5.9 (2.5–9.4)	< 0.001
Wound infection	49/44 (53)	5.13 (3.64–7.11)	< 0.001	10.6 (6.5–14.8)	< 0.001
Urological complication	15/65 (19)	1.60 (0.90–2.63)	0.067	–	–
Thromboembolic complication	12/20 (38)	3.68 (1.93–6.38)	< 0.001	1.5 (0.5–3.0)	0.042
Chyle leakage	6/32 (16)	1.48 (0.58–3.06)	0.318	–	–
Postoperative bleeding	24/11 (69)	5.52 (3.49–8.33)	< 0.001	5.7 (2.9–8.5)	< 0.001
Bowel injury	24/2 (92)	8.03 (5.03–12.30)	< 0.001	5.8 (3.1–8.6)	< 0.001

Postoperative surgical procedure under general anesthesia

*PAF* population-attributable fraction

^a^Multivariable poisson regression

^b^Logistic regression-based estimates of confounder-adjusted attributable fractions

**Table 7:** Risk-adjusted associations and population-attributable fraction between hospital readmissions and complications after elective resection for gastric cancer

Postoperative complication	No. with/without readmission (% readmission)	Risk-adjusted association^a^		Risk-adjusted PAF^b^	
		Adjusted relative risk (95% CI)	*p* value	PAF% (95% CI)	*p* value
Pulmonary complication	53/268 (17)	1.39 (1.01–1.88)	0.026	3.0 (– 2.9–8.9)	0.315
Anastomotic leakage	32/122 (21)	1.68 (1.12–2.43)	0.005	2.3 (– 2.2–6.7)	0.319
Cardiac complication	19/103 (16)	1.20 (0.72–1.89)	0.405	–	–
Acute delirium	20/83 (19)	1.52 (0.92–2.37)	0.044	1.1 (– 2.1–4.3)	0.508
Abscess	31/54 (37)	3.24 (2.16–4.70)	< 0.001	7.0 (3.2–10.9)	< 0.001
Wound infection	27/66 (29)	2.70 (1.75–3.99)	< 0.001	5.1 (1.5–8.8)	0.006
Urological complication	10/70 (13)	0.97 (0.48–1.74)	0.912	–	–
Thromboembolic complication	6/26 (19)	1.45 (0.57–3.01)	0.300	–	–
Chyle leakage	5/33 (13)	1.14 (0.40–2.49)	0.762	–	–
Postoperative bleeding	9/26 (26)	2.15 (1.02–3.97)	0.010	1.4 (– 0.6–3.5)	0.172
Bowel injury	9/17 (35)	2.93 (1.38–5.45)	< 0.001	1.4 (– 0.6–3.4)	0.164

Readmission to hospital within 30 days after initial discharge

*PAF* population-attributable fraction

^a^Multivariable Poisson regression

^b^Logistic regression-based estimates of confounder-adjusted attributable fractions

The risk-adjusted population-attributable fractions (PAF) of each complication-outcome pair are presented in Tables 3, 4, 5, 6, and 7. The PAF embodies the percentage reduction of a given outcome that is expected if that complication would be completely prevented (see also Supplementary File: Fig. 1a–e). Anastomotic leakage and pulmonary complications had the greatest overall impact on both postoperative mortality and prolonged hospitalization (PAF 29.2% [95% CI 19.3–39.1] and 12.9% [95% CI 9.7–16.0] for anastomotic leakage, respectively, and PAF 21.6% [95% CI 10.5–32.7] and 14.7% [95% CI 11.0–18.8] for pulmonary complications, respectively). Elimination of anastomotic leakage and intra-abdominal abscesses would have resulted in a reduction of re-interventions of 25.1% [95% CI 20.5–29.7] and 11.5% [95% CI 8.2–14.8], respectively. Again, anastomotic leakage, together with wound infections, had the greatest overall impact on reoperations (PAF 30.3% [95% CI 24.3–36.3] and 10.6% [95% CI 6.5–14.8, respectively). Intra-abdominal abscesses and wound infections were the complications with the greatest overall impact on hospital readmission (PAF 7.0% [95% CI 3.2–10.9] and 5.1% [95% CI 1.5–8.8], respectively). A large part of the causes of the study outcomes (grouped under ‘other’ factors in Supplementary File: Fig. 1a–e) cannot be specified, due to variation in the data that cannot be explained by patient and/or treatment characteristics and complications. Interestingly, these ‘other’ factors, also contributed to all clinical postoperative outcomes in large numbers, accounting for 32.9% of postoperative mortality, 50.8% of prolonged hospitalization, 32.5% of re-interventions, 27.3% of reoperations, and 78.7% of hospital readmissions.

## Discussion

In this population-based study, the clinically most relevant complications after gastrectomy for gastric cancer were evaluated in a Western population using the PAF. Anastomotic leakage and pulmonary complications were demonstrated to have the greatest overall impact on postoperative mortality, prolonged hospitalization, re-interventions, and reoperations. Intra-abdominal abscesses and wound infections also had a high impact on re-interventions, reoperations, and hospital readmissions.

The PAF is a measure with which the contribution of specific postoperative complications on a subsequent clinical outcome can be quantified [14–16]. It provides a perspective of prevention of disease actions considering the risk of disease in exposed individuals and the prevalence of exposure in the population. Thus, high risk of disease in exposed individuals can have low population impact if the risk factors associated with it are rare, whereas low risk may impact public health when exposures are frequent. In colorectal and esophageal cancer surgery, it already has been shown useful and helps to guide surgical quality improvement initiatives [14, 16]. For instance, an American study that assessed the PAF of complications after colorectal cancer surgery pointed out that the continued focus of federal quality initiatives on specific outcomes (e.g., urinary tract infections) had to be changed, illustrated by an estimated PAF of less than 10%, which indicates that its effect on the population is relatively minor [14].

The significance of complications such as anastomotic leakage and pulmonary complications after gastrectomy has previously been acknowledged in the literature [4, 7–9]. However, using the PAF, the current study allows us to confirm the impact of these complications for the first time. Pulmonary complications, being the most common complication in our study (15%), indeed demonstrated to be an important driver of postoperative mortality and prolonged hospitalization. General advises to prevent pulmonary complications include abstinence of smoking before surgery, pre-operative pulmonary rehabilitation and adequate pain management (using epidural analgesia and a transversus abdominis plane block). In addition, enhanced recovery after surgery (ERAS) programs could help to prevent pulmonary complications after major surgery [10, 29]. Future studies should focus on factors to prevent these complications, as this will reduce deteriorated outcomes with the PAFs mentioned in the current study.

Although the centralization of gastric cancer surgery has led to a reduction in the anastomotic leakage rate in The Netherlands [18–20], the manifestation of anastomotic leakage remains a frequently occurring and significant problem. At the same time, this study demonstrated that anastomotic leakage is a major driver of postoperative mortality, re-interventions, and reoperations. Our study indicates that preventing or managing anastomotic leakage should receive priority when developing or adapting surgical quality improvement programs. Initiatives to reduce the anastomotic leakage rate may include further centralization and adequate proctoring programs with hands-on courses for new surgeons [30]. A proctoring program, that allows beginning surgeons to operate together with an experienced surgeon during a reasonable amount of cases, can be a starting point for going through the learning curve of gastric cancer surgery. Data on the number of procedures required for completion are scarce and vary, with studies reporting between 10 and 100 procedures to complete the learning curve, depending on the outcome under investigation [31, 32]. In addition, pre-operative evaluation of a patients’ condition, for instance, the vascular status [33], may contribute to select and treat patient who are prone for developing anastomotic leakage. Moreover, if an adequate oncologic resection is possible, subtotal gastrectomy should be preferred over total gastrectomy, as the former results in a lower risk of postoperative complications and better quality of life [34, 35]. Finally, special intraoperative attention should be paid to the perfusion of and tension on the anastomosis and its staple-line technique (hand sewn versus stapler and linear versus circular) and reinforcement to avoid leakage and support the healing process [36, 37].

Both wound infections and abscesses had a high impact on re-interventions and reoperations, which highlights the importance of preventing these complications as well. The occurrence of wound infections is counteracted by pre-operative prophylactic administration of antibiotics. Furthermore, minimally invasive surgery could play a role in decreasing wound infections and pain [38], thereby also resulting in shorter hospitalization. In addition, the centralization of gastric cancer surgery and completion of the learning curve also play an important role, since intra-abdominal abscesses are often an expression of anastomotic leakage or caused by other perioperative complications.

This study demonstrated that a large part of postoperative outcomes can be attributed to postoperative complications. However, approximately one-third of the outcomes could not be attributed to patient and treatment-related characteristics or postoperative complications. For example, 78.7% of hospital readmissions could not be attributed to the well-defined complications or patients’ demographics. This finding indicates that further research is warranted to identify these other drivers that attribute to outcomes after gastric cancer surgery. This knowledge could then be used to modify these factors and thereby reduce the clinical deteriorated outcomes.

Although this is the first study to evaluate the clinically most relevant postoperative complication after gastrectomy, a few limitations should be discussed. First, unknown confounders and time-varying perioperative care may have affected the associations between postoperative complications and the clinical outcomes. Second, DUCA only registers data up to discharge and 30 days postoperative lacks data on long-term survival and makes no distinction in the different locations, where anastomotic leakage can occur, although it would have been interesting to evaluate different anastomotic sites. Moreover, unfortunately, no cost aspect could be included in this study, since data on costs are not registered in DUCA. However, the previous studies have shown that postoperative complications are the main drivers of costs of cancer surgery [39]. As such, the extrapolation of this knowledge to the current study indicates that pulmonary complications and anastomotic leakage are probably the main drivers of costs after gastrectomy. Finally, factors to prevent complications could not be evaluated in this study, as the DUCA lacks significant data to perform these multivariable analyses.

In conclusion, using the PAF for the first time in gastric cancer surgery, this population-based study identified anastomotic leakage and pulmonary complications as major attributors to clinical outcomes after gastrectomy. Surgical quality improvement programs that can successfully reduce these complications will have the greatest potential to reduce deteriorated outcomes.

## Electronic supplementary material

Below is the link to the electronic supplementary material.

Supplementary file1 (DOCX 325 kb)
