# Supplementary material for: Identification of the clinically most relevant postoperative complications after gastrectomy: a population-based cohort study
Source: Gastric Cancer. 2019 Sep 3;23(2):339–48. doi: 10.1007/s10120-019-00997-x (PMC7031165; doi:10.1007/s10120-019-00997-x)
Supplement: Supplementary file 1 — Supplementary file1 (DOCX 325 kb) [file 10120_2019_997_MOESM1_ESM.docx]

**
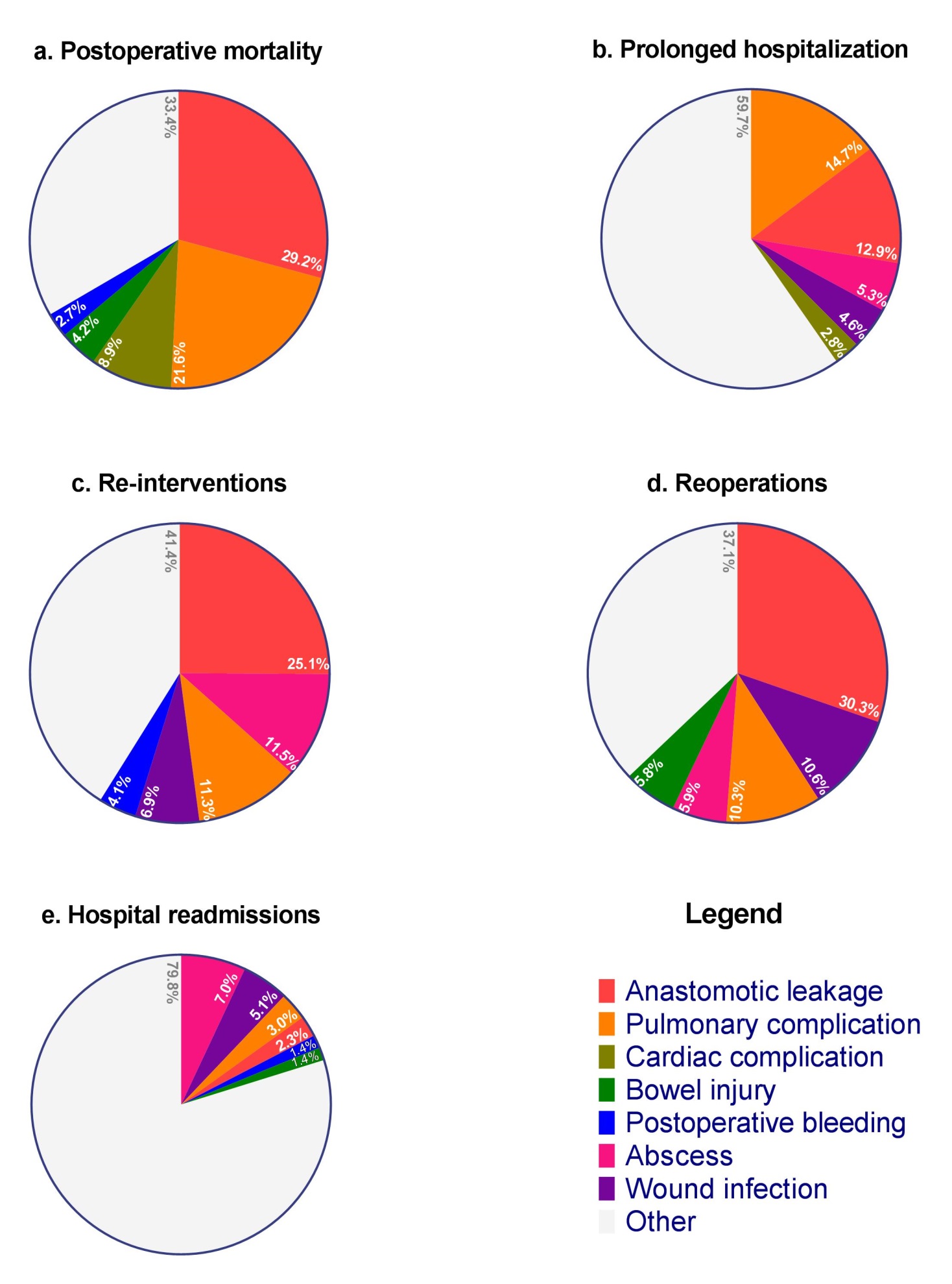
SUPPLETMENTARY FILE: Figure 1a-e**

**Figure 1** Risk-adjusted population attributable fractions for the complications having the greatest impact on each outcome
